# Survival in Patients with Relapsed-Refractory Multiple Myeloma: Indirect Comparison of Six New Treatments

**DOI:** 10.3390/hematolrep15010006

**Published:** 2023-01-13

**Authors:** Luca Cancanelli, Melania Rivano, Lorenzo Di Spazio, Marco Chiumente, Daniele Mengato, Andrea Messori

**Affiliations:** 1 Hospital Pharmacy Department, Azienda Ulss 2 Marca Trevigiana, Castelfranco Veneto, 31033 Treviso, Italy; 2 Hospital Pharmacy Department, Binaghi Hospital, 09126 Cagliari, Italy; 3 Hospital Pharmacy Department, S. Chiara Hospital, 38122 Trento, Italy; 4 Scientific Direction, Società Italiana di Farmacia Clinica e Terapia (SIFaCT), 20159 Milan, Italy; 5 Hospital Pharmacy Department, Azienda Ospedale, Università of Padova, 35122 Padova, Italy; 6 HTA Unit, Regional Health Service, 50136 Firenze, Italy

**Keywords:** relapsed-refractory multiple myeloma, overall survival, CAR-T, non-CAR-T agents

## Abstract

In recent years, new treatments have been studied for relapsed-refractory multiple myeloma (RRMM), including two CAR-T products and a variety of non-CAR-T agents. Since direct comparisons between these innovative treatments are not available, indirect comparisons can be of interest. Reconstruction of individual patient data from Kaplan-Meier graphs (e.g., according to the Shiny method) has been the subject of numerous reports that have fully validated their performance. In the present systematic review, we evaluated six treatments proposed for RRMM, including two CAR-T products (ciltacabtagene autoleucel and idecabtagene vicleucel) and four treatments not based on a CAR-T (melflufen plus dexamethasone, isatuximab plus dexamethasone, selinexor, and belantamab). The endpoint was overall survival (OS). Our results showed statistically significant differences in OS across these treatments. In particular, ciltacabtagene autoleucel showed better OS than idecabtagene vicleucel. As regards non-CAR-T treatments, the ranking in OS was headed by isatuximab plus dexamethasone, followed by belantamab, selinexor, and melflufen plus dexamethasone. In conclusion, while the Shiny method has confirmed its validity in reconstructing individual patient data, our indirect comparisons have offered some original clues to interpret the results of OS published in these studies.

## 1. Introduction

In patients with multiple myeloma (MM) who are triple-class exposed, treatment outcomes show an overall response rate of only 31% with median overall survival (OS) around nine months (Gandhi et al. 2019) [1]. To improve outcomes in this patient population, there is an unmet need for novel and innovative therapies.

Selinexor, an oral selective inhibitor of nuclear export (SINE), has been approved to treat patients who have received more than three previous therapies (Chari et al. 2019) [2]. Likewise, belantamab mafodotin, a B-cell maturation antigen (BCMA)-directed antibody and microtubule inhibitor conjugate, has also received approval to treat these patients (Lonial et al. 2020) [3]. Isatuximab is a CD38-targeting immunoglobulin (Ig)G1 monoclonal antibody approved in the US and EU in combination with pomalidomide and dexamethasone for the treatment of relapsed/refractory MM. The combination of isatuximab plus dexamethasone has been studied by Dimopoulos et al. (2021) [4] in a phase II trial conducted in this population of patients. Melflufen (or melphalan flufenamide), a peptide-drug conjugate that targets aminopeptidases and releases alkylating agents into tumor cells, is another agent that has been tested in combination with dexamethasone; the phase II HORIZON trial based on this combination was published in 2021 by Richardson et al. (2020) [5]. As regards the use of chimeric antigen receptor T (CAR T)-cell therapy in MM, ciltacabtagene autoleucel (also known as cilta-cel) is a product that expresses two BCMA-targeting single-domain antibodies designed to confer avidity, as well as a CD3 signaling domain with a 4-1BB costimulatory domain to optimize T-cell activation and proliferation; cilta-cel was firstly studied in a phase I trial (Wang et al. 2019) [6] and was then evaluated in a multi-center phase 1b/2 study, the CARTITUDE-1 trial (Berdeja et al. 2021) [7]. Finally, ide-cel is another CAR-T cell tested in patients with MM (Munshi et al. 2021) [8].

This overview of the current literature underscores the large number of treatments recently proposed for this disease condition. One important point is that all of these trials are phase II and, therefore, do not allow any direct comparison between these agents. In this framework, indirect comparisons can be made to synthetize the available evidence and to generate a comparative synopsis on effectiveness. All the above-mentioned trials have included OS among their clinical endpoints and have presented Kaplan-Meier curves to describe the survival pattern over time. This is an ideal context to apply a new technique of artificial intelligence (the Shiny method [9]) that reconstructs individual patient data from the published graphs of Kaplan-Meier curves. Reconstructed patient-level data allow one to carry out indirect comparisons by including multiple Kaplan-Meier curves within a single graph.

## 2. Methods

### 2.1. Literature Search and Selection of Pertinent Clinical Trials

**Figure 1:**
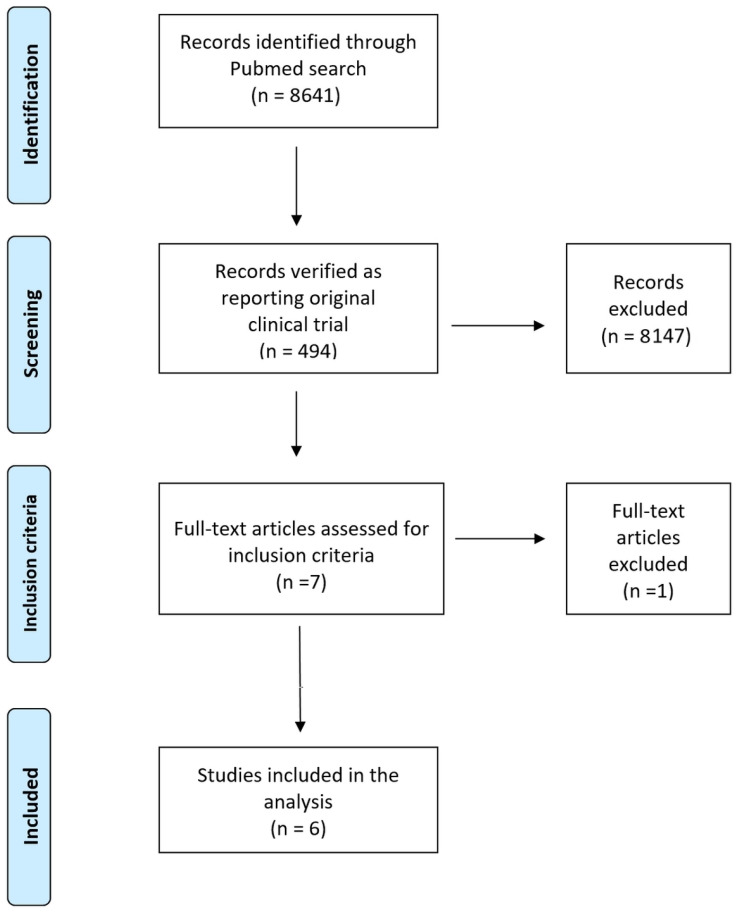
PRISMA flow chart summarizes the literature search.

In this systematic review, we carried out a search of the literature to identify the trials eligible for our analysis. This search was conducted in PubMed (last query on 31 December 2021) and covered the period from January 2019 to December 2021. A single search term (“multiple myeloma”) was employed, along with the filter “clinical trial”. Trial selection was handled according to the Preferred Reporting Items for Systemic Review and Meta-Analyses (PRISMA) approach [9]. We also searched the Cochrane Library for any recent systematic review on the subject, the ClinicalTrials.gov database, and the websites of the European Medicines Agency (EMA) and the U.S. Food and Drug Administration (FDA). The above keywords were also employed for these additional searches (Figure 1).

Our analysis included the trials that met the following criteria: (a) relapsed-refractory multiple myeloma; (b) phase-II or phase-III; and (c) determination of overall survival (OS) based on a Kaplan-Meier curve with a follow-up of at least one year.

To synthetize the information reported in included trials, we applied a quite new technique of artificial intelligence (the Shiny method [10]) that reconstructs individual patient data from the published graphs of the Kaplan-Meier curves.

### 2.2. Analysis of Kaplan-Meier Curves of Overall Survival

From each study, we analyzed the Kaplan-Meier graph of OS reported for the treatments under examination, along with the total number of enrolled patients and the total number of deaths. Information on disease condition at baseline was recorded, including the patients’ history and the number of previous lines of treatment. For each OS curve of each trial, we reconstructed patient-level data from by application of the Shiny method [10]. Firstly, the graph of each of the six Kaplan-Meier OS curves was digitalized and converted into x–y data pairs using Webplotdigitizer [11]; then, the Shiny package (Version: 1.2.2.0; subprogram “Reconstruct Individual Patient Data”; https://www.trialdesign.org/one-page-shell.html#IPDfromKM (accessed on 4 January 2023) [10]) was used to reconstruct patient-level data from x–y data pairs of the curve, total number of enrolled patients, and total number of events.

### 2.3. Generation of Treatment-Specific Kaplan-Meier Curves from Reconstructed Patient-Level Data and Statistical Comparison between Treatments

All of our analyses were based on the endpoint of OS. Our statistical comparisons between treatments were based on standard Cox analysis. Results of pairwise comparisons were expressed as hazard ratio (HR) with 95% confidence interval (CI). Medians for each treatment group (with 95% CI) were also determined. Covariate analysis was limited Resultsto the treatment given to individual patients (which was handled as a categorical variable). For these statistical analyses we used three packages (“coxph”, “survfit”, and “ggsurvplot”) under the R-platform [12]. Apart from treatment, other covariates were not available at patient level owing to the “reconstructed” nature of our analysis. The generation of pooled survival curves from reconstructed individual patient data has recently been shown to be a simple but efficient alternative to survival meta-analysis [12,13,14,15,16].

## 3. Results

### 3.1. Literature Search and Selection of Pertinent Clinical Trials

Figure 1 presents the selection process based on the PRISMA schematic that identified a total of six pertinent trials. The main characteristics of these trials are reported in Table 1, along with the treatments that were evaluated and compared in terms of OS. A total of six patient cohorts were subjected to the procedure of individual patient data reconstruction. Overall, six treatments had been given to these patient cohorts. These treatments were analyzed and compared with one another based on the endpoint of OS.

In conducting our analyses, we tried to avoid the inclusion of an excessive number of treatments in the same Kaplan-Meier graph. For this purpose, in cases where a phase-III trial clearly favored the treatment arm and identified no important role for the control arm, our Kaplan-Meier curve included only the treatment arm and not the treatment given to the controls. Finally, as shown in the PRISMA schematic, although the CANDOR trial was identified by our search of the literature, this trial could not be included because only progression-free survival (the primary endpoint) has thus far been reported, and not OS. This is because, in the CANDOR trial, while at present 140 deaths have occurred in the cohort of 312 patients, the analysis of OS will be carried out according to the study protocol when data are more mature (namely, when at least 230 deaths or 58 months after the first participant has been enrolled, whichever occurs earlier) [17].

### 3.2. Comparison of Four Non-CAR-T Treatments

**Figure 2:**
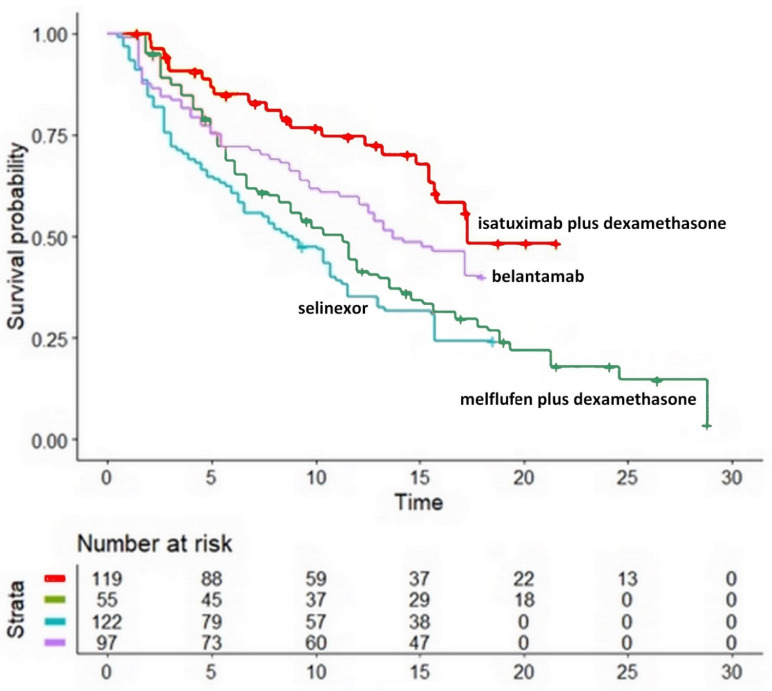
**Kaplan-Meier curves from reconstructed patient-level data.** These four survival curves were obtained by reconstruction of individual patient data from four trials: melflufen plus dexamethasone (HORIZON), isatuximab plus desamethasone (NCT01084252), selinexor (STORM), and belantamab (DREAMM-2). The cohort given melflufen plus dexamethasone consisted of 119 patients, (O-12-M1 N = 119); the cohort given isatuximab plus desamethasone consisted of 55 patients, (NCT01084252 N = 55); the cohort given selinexor consisted of 122 patients (STORM = 122); and the cohort given belantamab consisted of 97 patients (DREAMM-2 = 97). Symbols: isatuximab plus dexamethasone in red, melflufen plus dexamethasone in green, selinexor in blue, belantamab in purple; time is expressed in months.

The reconstructed Kaplan-Meier curves for the four non-CAR-T treatments are shown in Figure 2. The following differences in OS were found: belantamab: HR, 1.61; 95%CI, 1.00 to 2.60 (*p* = 0.05); selinexor: HR, 2.26; 95%CI, 1.44 to 3.54 (*p* < 0.001); melflufen plus dexamethasone: HR, 2.63; 95%CI, 1.67 to 4.14 (*p* < 0.001). All the above values of HR refer to isatuximab plus dexamethasone as a common comparator.

### 3.3. Comparison of Two CAR-T Products

**Figure 3:**
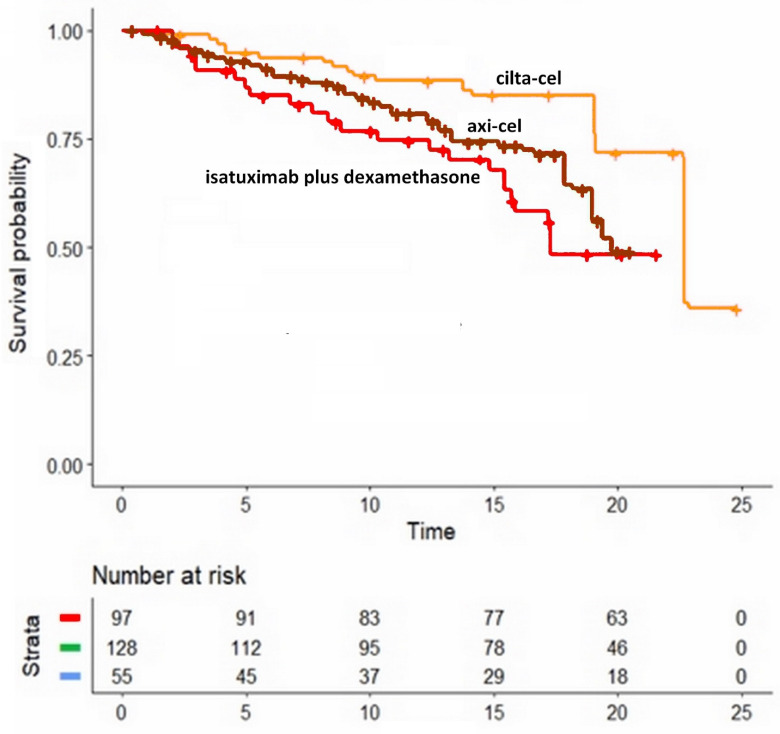
**Kaplan-Meier curves from reconstructed patient-level data.** These three survival curves were obtained by reconstruction of individual patient data from three trials: ciltacabtagene autoleucel (CARTITUDE-1), idecabtagene vicleucel (KarMMa), and isatuximab plus desamethasone (NCT01084252). The cohort given ciltacabtagene autoleucel consisted of 97 patients, (CARTITUDE-1 N = 97); the cohort given idecabtagene vicleucel consisted of 128 patients, (KarMMa N = 128); and the cohort given isatuximab plus desamethasone consisted of 55 patients, (NCT01084252 N = 55). Symbols: isatuximab plus dexamethasone in red, ciltacabtagene autoleucel in orange, idecabtagene vicleucel in brown; time is expressed in months. The curve for isatuximab plus dexamethasone is reported to make this figure more easily comparable to Figure 2.

The reconstructed Kaplan-Meier curves for the two CAR-T products are shown in Figure 3 (along with the curve for isatuximab plus dexamethasone, which is reported to make this figure more easily comparable to Figure 2). Ciltacabtagene autoleucel showed a significantly better OS compared with idecabtagene vicleucel (HR, 2.14; 95%CI, 1.34 to 3.42, *p* = 0.0014). The difference in OS favouring cilta-cel vs. axi-cel was statistically significant.

## 4. Discussion

This systematic review was aimed at presenting the current state of the art concerning a therapeutic issue where the rate of publication of new studies is high, and so the need emerges to offer a comprehensive picture of the most recent evidence. While these comparisons across different treatments cannot be directly transferred into practice, nevertheless, this type of narrative synthesis on this topic is worthwhile, mainly because of its high communication effectiveness. In the present work, the Shiny method has confirmed its most important advantage in that the timing of event occurrence (i.e., the length of follow-up) is accurately accounted. This does not occur with standard meta-analysis. On the other hand, a disadvantage of the Shiny method is that it loses the balance between treatment group and controls determined by randomization in individual trials. This disadvantage is less important when the clinical material is mainly represented by phase 2 trials.

The clinical material included in our analysis suffered from some heterogeneity. For example, the included studies spanned from phase 1b to phase 3 trials. Furthermore, the primary and secondary endpoints of all these trials essentially were objective response ratios, duration of response, and time-to-treatment failure, whereas our ex-post analysis was exclusively focused on OS. More importantly, the eligibility criteria differed to some extent across these trials (e.g., in the proportion of patients with refractory disease to one, two or up to five compounds). Likewise, the prior lines of therapy (which ranged from a median of two in the CANDOR trial, to six in the KARMMA/Cartitude-1 trial, and even seven in the DREAMM-2 trial) was another potential source of heterogeneity. Furthermore, one should keep in mind that the trial by Dimoplous et al. [4] evaluating isatuximab excluded prior anti CD38 exposure, which would be daratumumab in most cases. This is a potential confounding factor for our indirect comparisons because patients given isatuximab could be expected to fare somewhat better owing to this exclusion criterion; in other words, the efficacy of isatuximab-based treatment might have been over-estimated in our analysis because, in the absence of this exclusion criterion, the “true” efficacy of isatuximab could be slightly worse than that represented by our reconstructed survival curve. Despite these limitations, combining the different curves of OS into a single graph has generated useful information. In fact, the basic inclusion criteria across these trials were not the same (as previously pointed out), but anyhow, they were very similar. 

The main, well known limitation of the Shiny method is that this approach does not allow one to perform any multivariate analyses [18,19]. In more detail, while the covariate represented by the treatment can be adequately analyzed, no other variables beyond the treatment can generally be evaluated. In fact, while a Kaplan-Meier curve is the only source of information for the Shiny method, multivariate analyses are not generally presented according to Kaplan-Meier curves; hence, multivariate analyses can only be made using “true” patient-level data, and not using “reconstructed” patient-level data.

Numerous keys of interpretation are suggested by our results. As regards isatuximab plus dexamethasone, one potentially important advantage of this treatment is that its OS ranked first in the indirect comparisons made among non-CAR-T treatments. However, although this treatment was assumed to be a reference point in our analysis, it should be stressed that this combination is not currently considered a standard therapy. As regards belantamab, the patients given this treatment in the phase II trial were very advanced; hence, one advantage of belantamab is that treatment discontinuations due to toxicity were uncommon. Finally, selinexor and melflufen plus dexamethasone were ranked at the two worst positions according to our indirect comparisons based on OS. One should keep in mind that melflufen has recently been withdrawn by the FDA for emerging safety concerns from the phase III OCEAN trial; furthermore, the OCEAN trial, which has been published after the completion of our analysis, has shown that melflufen does not seem to provide any survival advantage, a conclusion that agrees with the findings of our comparative analysis.

This picture of effectiveness for both CAR-T and non-CAR-T treatments did not include the combination of carfilzomib, dexamethasone, and daratumumab, owing to the unavailability of the OS curve [17]. Since this curve is expected to become available in 2022, it will be worthwhile to include this triple therapy as the fifth arm in Figure 2 as soon as this information becomes available. Ranking this triple treatment in comparison with the others will be of great interest inasmuch as this treatment could soon be recognized as the new standard for refractory/relapsed MM. 

In this overall context, the high and deep response rates resulting from CAR-T cell products (cilta-cel and axi-cel) is an amazing and practice-changing fact that underscores a completely different therapeutic approach. These two CAR-T products ranked at the first two positions in terms of OS, and their better OS reached statistical significance compared with non-CART treatments. The only exception was the comparison between axi-cel vs. isatuximab plus dexamethasone, which did not reach statistical significance. Furthermore, it should be stressed that CAR-T cell treatments are intrinsically different from standard pharmacological treatments, and this may explain their relatively high effectiveness in cases in whom a low response to treatment could be expected [20].

In conclusion, the main finding generated by our analysis is the remarkably longer OS found for CAR-T products compared with non-CAR-T treatments. One strength of our analysis is the excellent performance of the Shiny method in reconstructing individual patient data from published Kaplan-Meier survival curves [10,13,14,15,16]. Its main weakness lies in the indirect nature of our comparisons, with all consequent implications already discussed in previous studies [18,19].

## Figures and Tables

**Table 1:** Characteristics of the six studies included in our analysis.

Trial	First Author, Year of Publication	Inclusion Criteria	Treatment Group (n/N)	Patients	Events
HORIZON	Richardson 2021 [5]	Patients had received at least two prior lines of therapy, including an immunomodulatory agent and proteasome inhibitor, and were refractory to pomalidomide and/or an anti-CD38 monoclonal antibody. RRMM was defined as disease that was nonresponsive while on primary or salvage therapy or progressed within 60 days of last therapy.	melflufen plus dexamethasone	119	10
NCT01084252	Dimopolous 2020 [4]	Eligible patients had MM refractory to both an immunomodulatory agent (IMiD) and a proteasome inhibitor (PI), or had been treated with ≥3 prior lines of therapy, including an IMiD and a PI. Patients had to have received an alkylating agent, achieved at least a minimal response to a prior line of therapy, and could have received prior stem cell transplant.	isatuximab plus dexamethasone	55	41
STORM	Chiari 2019 [2]	Eligible patients had measurable myeloma according to International Myeloma Working Group, had previously received treatment with bortezomib, carfilzomib, lenalidomide, pomalidomide, daratumumab, glucocorticoids, and an alkylating agent, and had disease refractory to at least one immunomodulatory drug, one proteasome inhibitor, daratumumab, glucocorticoids, and their most recent regimen.	selinexor	122	121
DREAMM-2	Lonial 2021 [3]	Patients with relapsed or refractory multiple myeloma with disease progression after three or more lines of therapy and who were refractory to immunomodulatory drugs and proteasome inhibitors, as well as refractory or intolerant (or both) to an anti-CD38 monoclonal antibody with an Eastern Cooperative Oncology Group performance status of 0–2, were recruited.	belantamab	97	97
CARTITUDE-1	Berdeja 2021 [7]	Patients with a diagnosis of multiple myeloma who received three or more previous lines of therapy or were double-refractory to a proteasome inhibitor and an immunomodulatory drug and had received a proteasome inhibitor, immunomodulatory drug, and anti-CD38 antibody	ciltacabtagene autoleucel	97	88
KarMMa	Munshi 2021 [8]	Patients with disease after at least three previous regiments, including a proteasome inhibitor, an immunomodulatory agent, and an anti-CD38 antibody were enrolled	idecabtagene vicleucel	128	101

## Data Availability

The source code for the R-language script implementing our analysis is available upon request.
